# Subscapularis Function After Total Shoulder Arthroplasty Using Lesser Tuberosity Osteotomy or Tenotomy

**DOI:** 10.5435/JAAOSGlobal-D-20-00032

**Published:** 2020-05-01

**Authors:** Patrick M. O'Brien, Jack E. Kazanjian, James D. Kelly, E. Rhett Hobgood

**Affiliations:** From Avera Medical Group Orthopedics and Sports Medicine, Sioux Falls, SD (Dr. O'Brien); Llanerch Orthopaedics, Havertown, PA (Dr. Kazanjian); San Francisco Shoulder Elbow and Hand Clinic, San Francisco, CA (Dr. Kell); and Mississippi Sports Medicine and Orthopaedic Center, Jackson, MS (Dr. Hobgood).

## Abstract

**Introduction:**

Subscapularis dysfunction is a recognized complication after total shoulder arthroplasty (TSA). However, optimal subscapularis management during TSA is controversial. Subscapularis tenotomy (ST) has been used, whereas lesser tuberosity osteotomy (LTO) has gained popularity. This study compares the clinical outcomes in patients undergoing TSA with either ST or LTO, focusing on subscapularis strength and overall function.

**Methods:**

Records were reviewed for TSA performed from 2010 to 2016 by a single surgeon at one institution. Patient age, sex, hand dominance, and the time of follow-up were recorded. Radiographs were obtained and interpreted. Range of motion was measured and the American Shoulder and Elbow Surgeons scores obtained. A graded belly-press test was used to determine the overall subscapularis function. Subscapularis strength was measured during a resisted belly-press maneuver. Statistical analysis was performed using a paired Student *t*-test or Fisher exact test, with *P* < 0.05 determining statistical significance.

**Results:**

Overall, 28 shoulders constituted the LTO group with 37 in the ST group. No difference was found regarding age, whether their surgical site was their dominant extremity, or the time to follow-up. Radiographically, all osteotomies went on to union, with one malunion noted. Range of motion was equivalent. No statistical difference was noted in subscapularis strength or in the American Shoulder and Elbow Surgeons scores. The overall subscapularis function also failed to show any notable difference.

**Discussion:**

In conclusion, either LTO or ST can be used during TSA to achieve successful clinical outcomes. The method of subscapularis management did not affect the subscapularis strength or overall function.

Surgical treatment of glenohumeral degenerative osteoarthritis with total shoulder arthroplasty (TSA) has been shown to be a reliable option.^1,2^ Takedown and mobilization of the subscapularis tendon is a requisite to gain access to the glenohumeral joint through the deltopectoral approach. Subsequently, subscapularis dysfunction after TSA has become a more commonly recognized complication.^3^ However, optimal management of the subscapularis during TSA remains controversial.

Currently, three options exist in subscapularis management. Traditionally, a subscapularis tenotomy (ST) was used, with a division created through the tendon which is directly repaired after completion of the procedure. A subscapularis peel (SP) can also be used, with the release of the tendon directly from its insertion on the lesser tuberosity and reattached via transosseous repair methods. These methods rely on a tendon-tendon and tendon-bone healing interface, respectively. Recently, a technique involving a lesser tuberosity osteotomy (LTO) has been described,^4,5^ with the thought that a bone-bone healing interface may potentially provide superior strength and the possibility of an accelerated recovery.

These different methods of subscapularis management have been tested biomechanically in previous investigations, with variable results observed.^6,7,8,9,10^ There have been several published studies currently comparing the clinical results of patients undergoing TSA with a LTO with those receiving either a peel or tenotomy.^11,12,13,14,15^ All repair methods involved some type of a transosseous suture construct, regardless of whether the tendon was released directly from its insertion or divided through the tendon substance. None of these previous studies had directly compared an LTO with a ST comprising an all suture, soft-tissue-only repair without the use of a transosseous inplant.

The purpose of this study is to compare the clinical outcomes in a nonrandomized, sequential patient population undergoing TSA by a single surgeon for primary glenohumeral osteoarthritis with either an LTO or ST. Study parameters include evaluation of postoperative complications, radiographic assessment, physical examination findings (specifically focusing on dynamic subscapularis strength and overall function), and validated outcome scores.

## Methods

After Institutional Review Board (IRB) approval, the records were retrospectively reviewed for all TSA performed from September 2007 to September 2016 at one institution by the senior author (E.R.H.). The first LTO was performed in 2010, so the study period was narrowed from January 2010 to September 2016 to obtain a more homogenous patient group. The specific method of subscapularis management, LTO versus ST, was determined intraoperatively at the discretion of the primary surgeon. Inclusion criteria included a diagnosis of primary glenohumeral osteoarthritis. Patients with a diagnosis of rheumatoid arthritis or osteonecrosis were excluded because their soft-tissue quality could be compromised. Any patients with a history of previous open shoulder surgery were excluded. Patients undergoing previous shoulder arthroscopy, however, were included in the study group. A one-year minimum follow-up was established.

Overall, 108 TSA were performed during the study period, with 38 using an LTO. Of these 38 shoulders, 7 did not meet the inclusion criteria, with 3 being unavailable for clinical follow-up. This provided 28 shoulders for the LTO study cohort. The remaining 70 shoulders had a ST performed. Of these, a similar sized group was randomly selected, with 37 available for clinical assessment.

All patients underwent TSA through a deltopectoral approach. A combination of DePuy and Tornier prostheses were used. Both systems used were third generation humeral implant designs with variability in inclination, version, and offset. The glenoid components were both all polyethylene with fluted central peg designs, allowing for ingrowth capability. All humeral components were inserted with a press-fit stem, with the glenoid component placed with cement in the peripheral peg holes and bone graft within the central peg hole. Surgical technique was identical for both groups except for the subscapularis management.

### Lesser Tuberosity Osteotomy Technique

As previously described, a deltopectoral approach is performed, with the cephalic vein identified and retracted laterally. The superior 1 cm of the pectoralis major is divided, and the rotator interval identified and released. The long head of the biceps is followed into the joint, and a tenotomy is performed. The anterior humeral circumflex vessels are identified and ligated.

Osteotomy is then performed at the base of the lesser tuberosity, beginning laterally, with an oscillating saw and completed with an osteotome, with the medial extent of the osteotomy exiting just lateral to the articular margin. A complete 360° release is performed around the subscapularis to aid in mobilization. A tagging suture is placed, and the subscapularis is packed medially. The humeral head is cut. A full release of the labrum and capsule is performed from the glenoid. The glenoid surface is prepared and the polyethylene implant placed. The humeral canal is then prepared and trial implant placed. If satisfactory, the final humeral implant is then assembled.

**Figure 1:**
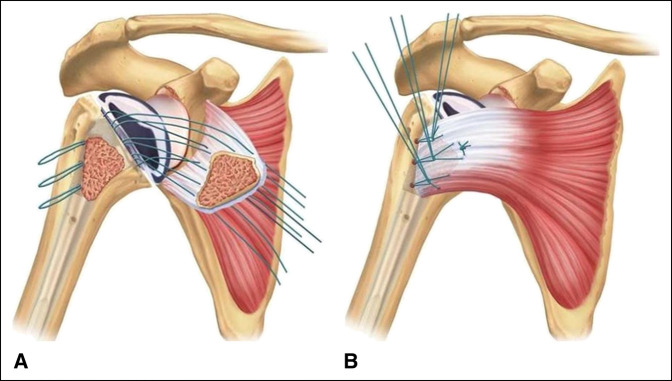
Illustration of the lesser tuberosity osteotomy repair technique after suture passage (**A**) and tying down in the “rack and hitch” fashion (**B**).

**Figure 2:**
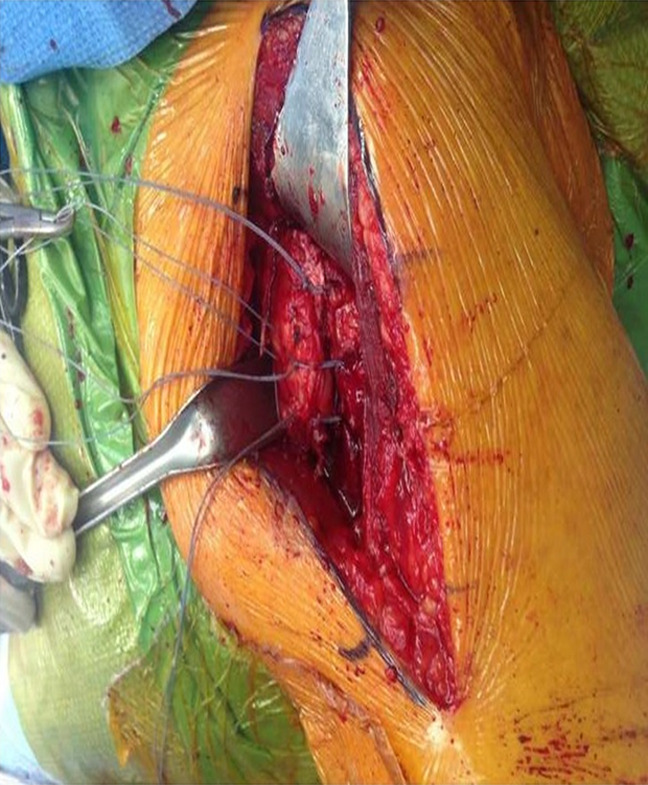
Intraoperative photograph showing the final lesser tuberosity osteotomy repair construct.

Before the final stem insertion, three drill holes are made just lateral to the bicipital groove, entering into the humeral canal. These are positioned at the superior, middle, and inferior aspects of the osteotomy. Looped No. 2 FiberWire (Arthrex) sutures are then passed through each drill hole with a suture passing device. An additional No. 2 FiberWire suture is also placed around the prosthesis, and the final implant is impacted into place. A free needle is then used to pass the suture ends through the subscapularis bone-tendon junction. Thus, a total of eight passes are made through the tendon. This is illustrated in Figure 1, A. The looped sutures are first tied in a “rack and hitch” fashion by forming a noose with the loop and then passing the two corresponding suture ends through the noose. The three “rack and hitch” sutures are individually tensioned and secured by throwing multiple half hitches on top of the noose. Last, the suture around the neck of the implant is tied. This is illustrated in Figure 1, B. An intraoperative photograph of the final repair construct is seen in Figure 2. The rotator interval is closed, a biceps tenodesis is performed into the pectoralis major repair, and the remaining wound is closed in a layered fashion.

### Subscapularis Tenotomy Technique

A deltopectoral approach is performed, as described previously. The subscapularis tendon is identified, and tagging sutures are placed at the superior lateral corner. A longitudinal tenotomy is performed 1 cm medial to the insertion on the lesser tuberosity. A complete 360° release is performed around the subscapularis to aid in mobilization. The subscapularis is then packed medially. A full release of the labrum and capsule are performed from the glenoid. The humeral head is exposed and cut. The glenoid surface is prepared, and the polyethylene implant is placed as previously described. The humeral canal is then prepared and the trial implant is placed. If satisfactory, the final humeral implant is assembled and inserted.

ST repair is then performed with a No. 2 FiberWire suture in three figure-of-eight suture throws. An overlying No. 2 FiberWire is placed in a running/locking fashion to both close the rotator interval and reinforce the previous repair. All suture passes go through tendon only, and no transosseous sutures are used, resulting in an all-soft-tissue repair construct. Biceps tenodesis is performed in the pectoralis major repair, and the wound is closed in a layered fashion.

### Postoperative Course

Patients were placed in a postoperative pillow sling. Postoperative antibiotics were administered, and the patients were admitted to the hospital for one to two nights. Pendulum exercise programs were initiated in the immediate postoperative period. The initial clinic visit occurs at 2 weeks after surgery, where passive and active-assisted range of motion exercise programs are begun; however, external rotation is limited to neutral and no active internal rotation is allowed. After the 6-week clinic visit, active motion and strengthening activities are begun, but again no subscapularis strengthening is allowed. Active subscapularis strengthening is begun at 3 months after surgery.

### Clinical Assessment

**Figure 3:**
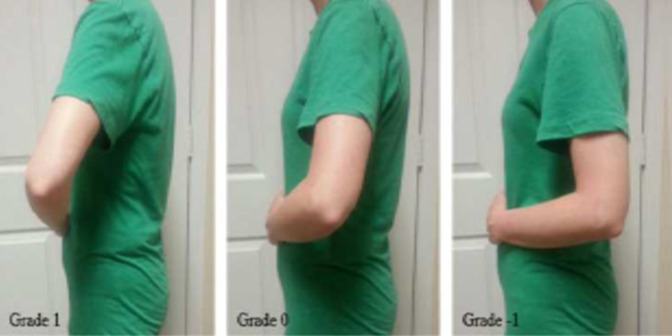
Photograph showing the description of the graded belly press test used to determine the overall subscapularis function. Grade is determined by the position of the elbow during the belly press maneuver. Grading numbering modified from that originally described by Jandhyala et al.^12^
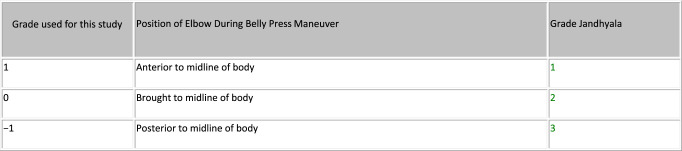

Patients were contacted and brought into clinic for evaluation. Their chart was reviewed for any postoperative complications. AP, scapular-Y outlet, and axillary radiographs were obtained at the time of their visit and assessed for any abnormalities. Patient age, sex, hand dominance, and the time to follow-up were recorded. Range of motion was measured with the use of a goniometer in three different planes (forward flexion, abduction, external rotation at the side). The American Shoulder and Elbow Surgeons (ASES) outcome scores were obtained. A graded belly-press test, modified in numbering from that described by Jandhyala et al^12^ and described in Figure 3, was then performed and used to determine the overall subscapularis function. A handheld dynamometer (Chatillon Systems) was used to determine the dynamic subscapularis strength. This was done with the patient performing a belly-press test, as described by Gerber et al,^16^ with the dynamometer strap placed around their wrist while actively pushing into their belly. The examiner then pulled the dynamometer away from the patient until their resistance was broken. The examination was performed three separate times and an average value recorded.

### Statistical Analysis

A statistical analysis of the measured variables was performed using an independent Student *t*-test assuming equal variance for continuous data and Fisher exact test for nominal data. An alpha level of 0.05 was used to determine statistical significance. Confidence intervals (CIs), using 95% grading, were calculated for the continuous data sets (the ASES and graded belly press).

## Results

There was a total of 61 patients (65 shoulders) included in the study. Four patients had bilateral TSA performed: three received a LTO on one side and a ST on the contralateral side, with one patient having a ST performed on both shoulders. This resulted in 28 shoulders included in the LTO group and 37 in the ST group.

Baseline characteristics between the two groups were recorded. No difference was found between the LTO and ST groups for age (68.8 years versus 64.8 years, *P* = 0.142) or whether their surgical site was their dominant extremity (17 dominant/11 nondominant versus 21 dominant/16 nondominant, *P* = 0.803). The time to follow-up was also similar between the groups, with the LTO group evaluated at an average of 28.4 months postoperatively and the ST group at 33.4 months (*P* = 0.194). A significant difference between the groups was detected for patient sex (LTO; 24 men/4 women versus and ST; 21 men/16 women, *P* = 0.015), with the LTO group containing a statistically significant greater proportion of male patients than the ST group. The patient demographics are summarized in Table 1.

**Table 1:** Summary of Patient Demographics Between Patients Receiving LTO and ST

Demographic	LTO (N = 28)	ST (N = 37)	*P* Value
Age	68.8 yr	64.8 yr	0.142
Sex	24 male/4 female	21 men/16 women	0.015
Operated on dominant extremity	17 dom/11 non-dom	21 dom/16 non-dom	0.803
Time to follow-up	28.4 mo	33.4 mo	0.194

LTO = lesser tuberosity osteotomy, ST = subscapularis tenotomy

No notable complications were noted during the postoperative course in the LTO group. One patient in the ST group developed a suture abscess that resolved with oral antibiotics and local wound care in the immediate postoperative period. Another patient in the ST group had continued pain and underwent a clinical workup to rule out infection, including serum inflammatory markers, bone scan, and joint aspiration. All aspects of the assessment were negative for infection, and the patient's pain was noted to improve.

Radiographically, all osteotomies went on to union. One malunion was noted. Two patients in the tenotomy group had a development of some heterotopic ossification. No radiographic evidence of humeral implant loosening was noted. Evaluation of the radiolucent lines behind the glenoid implant was not a focus of this study. However, no radiographic evidence existed of obvious glenoid implant loosening. No instances of instability or subluxation were seen.

The range of motion via measurement of forward flexion, abduction, and external rotation at the side also failed to show any significant difference between groups. The range of motion results are summarized in Table 2.

**Table 2:** Summary of Range of Motion Values for Patients Receiving Either LTO or ST

Arm Movement	LTO	ST	*P* Value
Forward elevation	156.6° ± 19.6°	157.0° ± 21.4°	0.845
Abduction	81.6° ± 21.8°	82.9° ± 19.2°	0.828
External rotation @ side	60.4° ± 16.1°	63.9° ± 14.6°	0.399

LTO = lesser tuberosity osteotomy, ST = subscapularis tenotomy

The overall subscapularis function, as determined by the graded belly-press examination, did not show any notable difference between the groups. There were 19 shoulders with a Grade 1 examination in the LTO group compared with 16 grade 1 findings in the ST group (*P* = 0.367). All of the remaining shoulders in the LTO group and ST group were a grade 0. Dynamic subscapularis strength, as measured by a handheld dynamometer during a resisted belly-press maneuver, was also comparable across the groups. The average strength value in the LTO group was 78.7 Newtons (95% CI: 69.3 N to 88.1 N), whereas it was 68.6 Newtons in the ST group (95% CI: 59.6 N to 77.7 N) (*P* = 0.166). Clinical outcome scores were also equivalent between the groups, with the average ASES of the LTO group 81.0 (95% CI: 73.9 to 88.4) and the average ST score 87.0 (95% CI: 82.4 to 91.6) (*P* = 0.203). The results are summarized in Table 3.

**Table 3:** Summary of Subscapularis Function (Determined by Graded Belly Press Test), Dynamic Subscapularis Strength, and Overall Shoulder Scores Between Patients Receiving Either LTO or ST

	LTO (N = 28)	ST (N = 37)	*P* Value
Grade 1	19	16	0.367
Dynamic strength	78.2 N	68.6 N	0.166
ASES score	81.0	87.0	0.203

ASES = American Shoulder and Elbow Surgeons, LTO = lesser tuberosity osteotomy, ST = subscapularis tenotomy

## Discussion

Optimal management of the subscapularis during TSA remains a controversial topic. The three commonly performed methods include a tenotomy, with an intratendinous division medial to the tendon insertion, a peel, with removal of the insertion directly off of the lesser tuberosity, and a LTO, with mobilization of a part of the bony tuberosity. These methods result in different healing interfaces, those of tendon-tendon, tendon-bone, and bone-bone, respectively. It is postulated that these different healing methods may result in repairs of varying strengths and could potentially influence surgical outcomes.

Several biomechanical studies have investigated the difference in the overall construct strength of these methods, with varied results. Giuseffi et al^6^ found markedly less cyclic displacement in cadaveric shoulders undergoing ST than those using an osteotomy, with no difference in the ultimate load to failure. However, in their cadaver model, Krishnan et al^7^ determined that lesser tuberosity fleck osteotomies were markedly stronger than a tenotomy repair. Ponce et al^8^ also found less cyclic displacement and a higher load to failure with LTO compared with both a tenotomy and transosseous repair. Van Thiel et al^10^ found no differences in the maximum load to failure or mode of failure between an osteotomy, a tendon-bone construct using bone tunnels, and a combined construct adding tendon-tendon fixation to the tendon-bone construct. Van den Berghe et al^9^ determined an improved failure rate under cyclic loading when comparing both bone-bone and tendon-tendon repairs with a tendon-bone construct. Given these differing results of initial construct strength, no one method of subscapularis mobilization can be deemed biomechanically superior.

Several clinical investigations have focused on the subscapularis function after TSA with different subscapularis management techniques. Jackson et al^17^ evaluated a tendon-tendon repair model with ultrasonography and determined that 7 of 15 patients had a rupture of the repair at the 6-month follow-up. Consequently, patients with subscapularis failure were found to have markedly lower Disabilities of the Arm, Shoulder and Hand (DASH) scores. Conversely, Armstrong et al^18^ reported 26 of 30 patients with an intact subscapularis after a tenotomy. Miller et al^3^ found a high proportion of patients with abnormal subscapularis function after a tendon-bone repair through bone tunnels, as determined by the results of the lift off and belly-press tests, as well as their ability to tuck in their shirts. In a subsequent study from the same institution, Qureshi et al^19^ measured the same parameters in a group of patients who underwent TSA using a LTO. When comparing these results with those in the previous study by Miller et al,^3^ an improvement was found in the LTO group in both belly-press testing (66.6% abnormal with tendon-bone versus 40% abnormal with LTO) and being able to tuck in a shirt (68.2% with difficulty with tendon-bone versus 16.6% difficulty with LTO).^19^ The authors determined that LTO provided superior results compared with the tendon-bone repair. In a retrospective review of patients undergoing LTO, Krishnan et al^7^ found a normal lift off test in 79% and a normal belly-press in 86% of patients, with 82% able to tuck in their shirts.

Direct clinical comparison of the different methods was first performed by Scalise et al,^15^ who compared clinical, radiographic, and ultrasonographic results in 15 shoulders undergoing tenotomy with repair through bone tunnels (tendon-bone model) and 20 receiving a LTO. All osteotomies were found to heal radiographically, with the LTO group showing fewer abnormalities on ultrasonography. The Penn Shoulder Scores were significantly higher in the LTO group at 1 year, but not different at 2 years. Internal rotation strength of the surgical extremity was also measured, with no difference noted when controlled for patient sex.

Two separate investigations performed by Lapner et al compared LTO with a SP. In one study, no difference in healing rates or fatty infiltration of the subscapularis was noted between the groups on postoperative CT imaging. In addition, no difference in subscapularis strength, ASES, and Western Ontario Osteoarthritis of the Shoulder (WOOS) Index scores was found. Interestingly, both methods showed an increase in fatty infiltration when compared with the preoperative state.^14^ In an earlier prospective, randomized controlled trial involving 43 LTO patients and 44 SP patients, it was again noted that no difference was found regarding the WOOS and ASES scores. Subscapularis strength, as measured through a belly-press test, was also found to be equivalent.^13^ Currently, this remains the largest patient population studied directly comparing two different methods.

Jandhyala et al^12^ compared 26 patients undergoing LTO with 10 receiving tenotomy. The tenotomy repair was performed in a dual row manner, with the more lateral row using a tendon-tendon suture and the medial row sutures placed through an intraosseous drill hole. Their main determination of subscapularis function was the graded belly-press test, which was modified in numbering and used in this study (Figure 3). They noted a significant improvement (*P* = 0.026) in patients with a grade 1 belly-press test in the osteotomy group (19 of 26) compared with the tenotomy group (3 of 10).

Most recently, Buckley et al^11^ retrospectively reviewed a group of 32 SP and 28 LTO patients. Subscapularis strength, as measured by resisted belly-press and bear hug tests, was not found to be different. The WOOS, DASH, and Constant scores were also equivalent. Ultrasonography assessment of both groups revealed four abnormalities in the SP group (three attenuated, one ruptured) and no abnormal tendons in the LTO group. Of note, the time to follow-up was markedly increased in the SP group.

The results in this investigation largely confirm the findings of these previous studies, with no statistically significant difference seen regarding the ASES clinical outcome scores or dynamic subscapularis strength. The overall subscapularis function, as measured by the graded belly-press test previously described (Figure 3), also failed to show a notable difference between groups, which is in conflict with the results reported by Jandhyala et al.^12^ Although an ultrasonography assessment was not performed in this study, radiographic findings were noted to be consistent with the previous findings, as all osteotomies went on to union, with one malunion noted. This study, however, differs from the previous studies in the repair method used for the tenotomy group. All of the previous clinical studies incorporated intraosseous suture passage, thus using a tendon-bone model; the repair model in this study was entirely soft tissue, using a tendon-tendon model. Thus, this is the first study in the literature that we are aware of comparing a bone-bone construct with a true tendon-tendon repair.

Limitations of this study include its retrospective nature and that the authors also served as clinical examiners and were not blinded to the patient group, which could introduce bias. In addition, patients were not randomized at the time of surgery, with the method of subscapularis management determined intraoperatively at the discretion of the primary surgeon. Study strengths include the fact that all surgeries were performed by a single surgeon during a relatively short period, allowing for a consistent surgical technique between the patients.

In conclusion, either LTO or ST can be used during TSA to achieve successful clinical outcomes, with no differences noted in the range of motion or ASES scores. The method of subscapularis management used also did not affect dynamic subscapularis strength or overall function.
